# A qualitative study of facilitators and barriers to participate in a needle exchange program for women who inject drugs

**DOI:** 10.1186/s12954-020-00425-9

**Published:** 2020-10-22

**Authors:** Malin Värmå Falk, Susanne Strömdahl, Anna Mia Ekström, Martin Kåberg, Niklas Karlsson, Helena Dahlborn, Anders Hammarberg

**Affiliations:** 1 Stockholm Needle Exchange, Stockholm Centre for Dependency Disorders, Stockholm, Sweden; 2 Department of Medical Sciences, Section of Infectious Diseases, Uppsala University, Uppsala, Sweden; 3 Department of Global Public Health, Karolinska Institutet, Stockholm, Sweden; 4 Department of Infectious Diseases, Karolinska University Hospital, Stockholm, Sweden; 5 Division of Infection and Dermatology, Department of Medicine Huddinge, Karolinska Institutet, Karolinska University Hospital Huddinge, Stockholm, Sweden; 6 Department of Monitoring and Evaluation, Public Health Agency of Sweden, Stockholm, Sweden; 7 Centre for Psychiatry Research, Department of Clinical Neuroscience, Karolinska Institutet, Stockholm, Sweden; 8 Stockholm Centre for Dependency Disorders, Stockholm Health Care Services, Stockholm County Council, Stockholm, Sweden

**Keywords:** Harm reduction, Women who inject drugs, Needle exchange program, Sexual and reproductive services, Injection drug use, Needle syringe program

## Abstract

**Background:**

Women who inject drugs (WWID) show higher levels of injecting risk behaviour compared to men, putting them at risk of contracting HIV and hepatitis C (HCV). Compared to men, WWID are also less present in harm reduction programs such as needle exchange programs (NEP). The aim of this study is to investigate reasons for, and barriers to, participation in NEP among WWID in Sweden, and to identify measures that could be taken to strengthen the program and increase participation among WWID.

**Method:**

In-depth interviews (IDIs) were conducted with 20 WWID who had participated in the Stockholm NEP for at least six months and was over 18 years old. IDIs were audio recorded and transcribed *et verbatim*. Qualitative content analysis was used to identify themes.

**Results:**

The need for sterile injection equipment was identified as the main driver to join and remain in the NEP program. Continuous participation in the NEP was further driven by easy access to a multitude of health-related services. The most valued service was the sexual and reproductive health services (SRHR), allowing participants to access contraceptives, cervical cancer screening and sexually transmitted infections testing (STI-testing). NEP staffs’ respectful treatment of participants further contributed to program participation. However, participants also expressed a number of concerns around NEP participation, which created barriers to joining. These included losing custody or visitation rights to children, male partner jealousy and violence, unwillingness to spend time in the waiting area and fear of receiving positive HIV/HCV test results. Practical barriers included limited opening hours and travel distance to the NEP. To strengthen the program, most participants requested additional SRHR services. Most participants also proposed some form of “women only” access to the NEP, to strengthen the feeling of the NEP as a safe space.

**Conclusion:**

This study identified factors that may increase uptake of NEP among WWID. Additional SRHR services and “women only” access are recommended to be implemented and evaluated as part of NEP. These findings may inform and improve the current scale-up of NEPs in Sweden to ensure equal access to services.

## Background

Injection risk behaviours, i.e. sharing of unsterile injection equipment, significantly contribute to maintain the high prevalence of hepatitis C (HCV), HIV and hepatitis B virus (HBV) infections among people who inject drugs (PWID). Several studies have concluded that women who inject drugs (WWID) show higher levels of injection risk behaviours compared to men [1–6], a difference also noted in the Scandinavian context [7, 8]. Higher injection risk behaviours among women may be explained by social, contextual and behavioural factors [1, 9]. In addition, societal norms and gender inequalities further exacerbate women’s vulnerability and perpetuate risk behaviour in terms of both injecting and sexual risk behaviours for hepatitis and HIV, as well as willingness among WWID to engage in prevention measures and care [4, 10–13].

To mitigate the harms of injecting drug use, several recommendations have been made [14, 15] including the scale-up of harm reduction (HR) services, which include services that focus on mitigating possible harms when injecting drugs while accepting the behaviour. Needle exchange programs (NEP), also called needle syringe programs, are one example of a HR program. NEP programs are unevenly distributed worldwide and WWID are less likely to participate in NEP compared to their male counterparts [16]. In Sweden, women constitute approximately one quarter of NEP participants, which suggest an under-representation. However, the exact size and gender distribution of the population of PWID in Sweden is unknown [7, 17]. Several explanations have been suggested, such as perceived stigma in being a WWID at harm reduction facilities, lack of confidentiality and low trust in healthcare providers, who often lack knowledge regarding WWID’s specific needs [18, 19]. In accordance with Swedish law, NEP participants must show identification at admission, which may be an additional barrier.

Access to HR interventions and NEP in particular has historically been inadequate in Sweden compared to most other countries. Despite being pioneers in implementing both NEP and opioid substitution treatment (OST) in the 1980s, this development was halted for political reasons, and NEPs were not scaled-up until 2017 [20]. Sweden is currently in a process of NEP expansion to achieve full national coverage. This expansion is an important part of the strategy to achieve UNAIDS and WHO targets to eliminate hepatitis and HIV by 2030 [15]. As part of this, however, it will be critical to increase program participation among WWID. Therefore, it is important to tailor interventions in order to reach a larger proportion of WWID and optimize retention in the program to ensure equality in access [21].

Few studies, however, have investigated facilitators and barriers for WWID regarding access to HR services. This is particularly true when investigating this field from WWID’s own perspective [18, 19, 22–24], which is vital in order to optimize the content of NEP.

The aim of this study is to fill this gap and utilize in-depth interviews (IDI) with WWID to investigate what facilitates WWID’s participation in a NEP, what the barriers to participation are, and the improvements that could be made to the program that would potentially motivate more WWID to participate in NEP.

## Method

### Study setting

The Stockholm NEP opened in April 2013 and (as previously described in detail [7, 20, 25]), offers sterile injection equipment (needles, syringes and paraphernalia) and testing for hepatitis A (HAV), HBV, HCV and HIV at inclusion [7]. In addition, the NEP also provides vaccination for HBV, naloxone pick-up, risk-reducing counselling, treatment for infectious diseases such as HIV and HCV, referrals to social services and substance use disorder clinics, including OST. SRHR services are also provided at the NEP by a midwife on certain weekdays. These services include counselling, contraception, STI-testing and cervical cancer screening. The NEP is staffed by physicians and nurses specialized in infectious diseases and psychiatry/substance use disorders, counsellors and midwives. The NEP is open on weekdays between 10 am and 4 pm (6 pm once a week), with no special opening hours for women.

### Data collection

IDIs were conducted between August 2018 and June 2019. Using a convenience sampling approach [26, 27] WWID who were at least 18 years old and had attended the NEP for at least 6 months were asked if they were willing to participate when coming to the NEP. Data saturation was assessed continuously by evaluating already collected data regarding repetition of themes to ensure that the research questions could be answered, when data saturation was achieved no more inclusions were made. IDIs were conducted by three trained interviewers and were held in a private room at the NEP to ensure privacy and confidentiality. The IDIs were guided by a semi-structured interview guide developed by the researchers guided by a conceptual framework to assess different aspects of participation in the NEP including questions regarding which NEP services participants had used. The conceptual framework based on access to healthcare and findings from previous quantitative studies of the cohort at this specific NEP informed the semi-structured interview guide used, while the analysis used the pre-defined themes from the guide as well as allowed for emerging themes within the data [27].

Written informed consent was obtained by all participants prior to the IDI. The participants received a 100 SEK (approximately 10 Euro) gift card at a local food store as reimbursement for their participation. Ethical approval for the study was obtained from the Regional Ethical Review Board in Stockholm, Sweden (2018/904-32).

### Data analysis

Interviews were performed in Swedish, recorded and transcribed *et verbatim*. Most interviews lasted 60 min, but the duration varied from 30 to 90 min. The data were analysed through a process guided by latent content analysis [26]. Transcripts were read repetitively, then independently analysed by two researchers assigning codes. These codes were then organized into themes including both the pre-defined areas in the semi-structured interview guide and new emerging themes (SS and MVF). Coded transcripts were then compared, and any discrepancies were resolved through discussion with a senior researcher (AH). Final themes were assigned in a discussion process between authors (MVF, SS, AH, AME). The quotes presented here were translated to English by a bilingual translator and reviewed by both the researchers and another bilingual translator.

### Participant characteristics

20 NEP WWID participants were included in the study. Half of them reported amphetamine as their main drug of injection, while the other half reported opioids, mainly heroin. Four participants received OST at the time of the interview. The age of the participants ranged between 25 and 62, with a median age of 40. Most participants reported previous exposure to HCV infection (*n* = 17), while only a few participants were living with HIV (*n* = 3). The majority reported temporary housing (social services accommodation or similar) or homelessness (*n* = 12), whereas eight had permanent housing. The median duration of NEP participation (stated by the participant) prior to the interview was approximately 3, 5 years (range 1–5, 5 years.).

## Results

### Factors serving as facilitators or barriers for NEP participation

The study identified facilitators, barriers and improvements to NEP as particular areas of research interest. This focus was also reflected in the interview guide. During the IDIs, and the subsequent data analysis, several aspects pertaining to these topics emerged. The findings are presented below as themes, categories and examples of quotes in Table 1.

**Table 1:** Areas, themes, categories and examples of quotes

Area	Theme	Category	Example of quote
Facilitators	Easily accessible services	Sterile injecting equipment SRHR services Social support and counselling HIV and HCV testing and treatment	*“…much help available under one roof that you would otherwise not get access to”* *”It has made a huge difference for many, for example receiving treatment for hepatitis C”*
	Staff’s approach	Respectful treatment Practical support	*”I have never felt ashamed or anything here, they [the staff], have helped me (…) get in contact with social services”*
Barriers	Fear of negative consequences	Fear of losing custody of children Fear of male partner jealousy and violence Fear of the waiting area Fear of being identified as a person who use drugs Fear of utilizing health care services due to previous bad experiences Fear of testing with the risk of being infected by HIV or hepatitis	*“I know two people who do not want to come here because they have under-aged children”* *“My boyfriend and I argue almost every time after I have visited the NEP, He is like: ‘who did you meet?!’ I am like ‘are you going to be jealous for that too?’ I can imagine that’s what it’s like for women who have men who don’t want them to come here [the NEP]”*
	Practical issues	Geographical distance Opening hours	*“I think it is a pity that it [the NEP] is only open a bit longer once a week. I have had trouble getting here in time”*
Improvement suggestions	Women only NEP	Opening hours for women only A site only for women	*”Open a women’s clinic”*
	Specific needs for women	Extended sexual and reproductive health services Education on sexual health Pregnancy and maternity groups	*“In general I think that asking more questions about birth control methods and such is important”*
	Practical improvements	Extended opening hours Mobile/shelter units Improved information regarding NEP	*“there should be more information about the needle exchange programme at addiction treatment services”*

## Facilitators for participation

### Easily accessible services

The reason for the initial visit to the NEP was the same for all participants: access to sterile injecting equipment. Participants expressed an increased use of sterile injection equipment following admission to the NEP, with a perceived decrease in own risk of contracting HIV and HCV, which in turn motivated continued participation in the program.

“It has meant a lot to me as I don´t have to keep on using the same old equipment and that the risk of contracting HIV or hepatitis C now is much lower”

Following access to sterile equipment, the easy access to a range of other services was highlighted by participants as the key facilitator to join and remain in the NEP. Services that were mentioned as valued were, for example, health interventions such as doctors’ appointments, HIV and HCV testing, HCV treatment, appointments for SRHR services, vaccinations, naloxone pick-up, counselling and wound care by the nurses.

Access to SRHR services emerged as particularly critical. Several participants expressed concern about not having visited a midwife or a gynaecologist in a long period prior to NEP participation and stated that the access to SRHR services at the NEP was their only access to SRHR services, including contraceptives and cervical cancer screening.

The access to these different services highlighted above was, combined, identified as the major common facilitator for participation, as expressed by this participant:

Everything from picking up syringes to meeting the midwife, meeting doctors to following up on abscesses

### NEP staffs’ respectful treatment of the participants

The staffs’ respectful treatment of participants was raised as another important reason for program retention. “Caring” as well as “encouraging” are examples of how the participants described the staffs’ attitude. Some participants described the NEP as the only place where they are treated with respect and without stigmatization, something that is highly appreciated.

“I am treated like a real person and I feel listened to”

Another appreciated aspect of the staffs’ work was their ability to support and aid the participants regarding reminders of appointments with other healthcare services, the social services and medication intake such as HIV treatment or antibiotics. One participant described how the staff encouraged and reminded her about the importance of testing for infectious diseases.

They [the staff] have like encouraged me, otherwise I would never have taken those tests

## Barriers for participating in the NEP

Several barriers for participating in the NEP were identified, as presented in Table 1. These barriers regarded both the initial visit and the frequency of continued participation at the NEP. These barriers were either experienced by the participants themselves or told to them by other WWID not participating in the NEP.

### Fear of negative consequences due to NEP participation

#### Fear of losing custody of children

More than half of the participants described the fear of losing custody of children as one barrier for participation in NEP. In Sweden, when healthcare professionals suspect that a child is neglected due to inadequate care, they have a legal duty to report to social services. Social services then decide if any actions ought to be taken to protect the child. This could include actions such as re-homing or restricting parental visitation rights. Throughout the study, several participants expressed a fear that reports to social services would result in immediate loss of custody or visitation rights of their children. Participants noted that this fear had created a barrier to initially visit the NEP, and also contributed to less frequent visits once they had joined the program. Several participants also raised this fear as a key factor why friends in similar positions refrained from joining in the NEP. Moreover, this barrier was also considered by participants as difficult to compensate for by any of the reasons defined as facilitators for participation, such as access to sterile injection equipment.

Because they—people who have children—cannot be anonymous. Social services have to be informed, and that causes resistance. I think this is why many women, single mothers and such, who have custody of their children, do not want to come.

#### Fear of male partner jealousy and violence

For some WWID, one identified barrier to visit the NEP is the fear of their male partner’s jealousy. Some participants reported situations where male partners hindered or did not allow their partner to visit the NEP. Reports were made that visits could cause outbursts of jealousy, reprimands or violent behaviour. Some participants reported that male partners visited the NEP in their female partner’s place.

Their guys don’t let them come here, in case they may meet someone else. It could be something like ‘No, I go, you can stay at home’, I can imagine something like that. Because there are quite a lot of guys [here]. It becomes difficult for those who can't handle their jealousy.

#### Concerns regarding the waiting area

The waiting room and the area just outside the clinic was regarded as a barrier to come to the NEP due to concerns of being harassed or threatened by male visitors who were also visiting the NEP. Experience of sexual and non-sexual violence was commonly reported among participants and the fear of meeting former perpetrators in the waiting area created a barrier to come to the NEP.

Well, it is probably the group of people, both in the waiting room and outside, that can be a little difficult at times. Maybe there are people you don’t want to meet or who you aren’t on good terms with, or who are very intoxicated.

Some participants also reported that they were bothered by the sale of drugs by others visiting the NEP and stated that they were experiencing a general feeling of discomfort in the waiting area. For instance, some participants expressed negative experiences with the waiting area being crowded. One participant expressed that she had refrained from entering the NEP due to the crowding and the anxiety it caused her.

I have left once when it was too crowded in the [waiting] room, I just left. I couldn’t handle it

#### Fear of being identified as a person who use drugs

Being identified as a person who use drugs was also raised as a barrier during IDIs. For some participants, the barrier was mainly about self-image, as entering the NEP reinforced a self-perceived stigma of being a person who use drugs.

It is taboo I think. I think it is shameful to come here after all

For other participants, the barrier of identification revolved more around the fear of losing anonymity and not trusting that their confidentiality would be upheld. This was expressed as a common belief that was held prior to the first visit to the NEP. Fear of being registered in different records was also among participants’ concerns.

It has to do with anonymity. Not with regards to the staff here, but in general, that you get registered…

#### Fear of healthcare services due to previous negative experiences

Lack of trust in public authorities and previous negative experiences when visiting healthcare facilities were repeatedly expressed by participants as barriers towards participating in the NEP. Several participants described experiences of being maltreated compared to other patients, for example that personnel at hospital wards would not answer calls from the room in which the participant was hosted. One participant explained how she felt when entering healthcare facilities in general in the following manner:

Yes, it is a bit like… you have your tail between your legs. You are always treated in that way—especially within healthcare—like you are a less worthy patient

#### Fear of blood testing and discovering that you are living with HIV or HCV

Another barrier identified to visiting NEP is the fear of finding out one’s HIV and HCV status. Participants were aware of the risk of acquiring these infections and worried about the consequences of testing positive. This fear was exacerbated by a lack of knowledge of available treatment if infected.

Of course I was a bit scared when I started coming here and was going to be tested for the first time in a very long time—what if I had HIV?

### Practical barriers for NEP participation

#### Geographical distance

The main practical barrier to NEP participation was the geographical distance to the NEP site, which at the time of this study was the only formal NEP within the Stockholm region. The distance was expressed as a barrier regarding the initial visit to the NEP as well as the frequency of visits once admitted to the program. When asked if she knew others who inject drugs and do not attend the NEP, one participant stated that the reasons as to why was:

The distance is too far [to the NEP] for those that I know

#### Opening hours

The majority of the participants also experienced limited opening hours as a barrier. Arriving when the NEP was closed sometimes resulted in a lack of sterile injection equipment until the next opening day.

### “If I could make a wish”—suggestions for improvement of the NEP

The following aspects emerged as key themes through the analysis and are identified as key aspects to focus on to strengthen NEP and potentially achieve a wider uptake amongst WWID (Table 1).

#### Addressing specific needs for women—sexual and reproductive health

Most participants expressed that services specifically designed to meet women's needs would attract more WWID to the NEP. As previously mentioned, SRHR services were appreciated, and increasing access to these services was suggested. Additional SRHR services and counselling was requested, for example education in sexual health combined with counselling from the staff regarding sexual risk behaviour and prevention. Some participants also expressed a need for maternity groups and special support, both during pregnancy and post-partum.

I would like some kind of community especially for mothers

#### A NEP for women only

Many participants expressed a desire for a NEP only for women and suggested that this would attract more women to the NEP. An alternative suggestion involved having scheduled hours at the NEP exclusively for women. Participants suggested a few hours every day or one day a week.

When only women are here, maybe more will get the courage to come

#### Practical improvements

Request for more generous opening hours in general was expressed in the IDIs, especially during the weekends in order to have access to sterile injecting equipment throughout the whole week. Furthermore, the opportunity to access the NEP more easily, for example as a mobile-unit or a new NEP site in another part of the region, was raised as a key factor that would improve the service.

Lastly, many of the participants expressed a lack of information regarding available services at the NEP within the social services, OST clinics, primary healthcare facilities, hospitals and at the police. The participants suggested that additional distribution of information about the NEP and its services could be a pathway to attract more women to the NEP.

…you should consider to really inform the girls that there are midwives working here, and that access to testing for chlamydia, pregnancy and so on is provided

## Discussion

Drawing on WWIDs’ experiences in Stockholm, Sweden, this study investigated facilitators, and barriers to join and remain in NEP, as well as the aspects that could be improved to strengthen WWID participation in the program. The main facilitator for entering the program was to access sterile injection equipment. Participation in the NEP was further facilitated by the easy access to a range of other services such as counselling and SRHR services. The encouraging and caring treatment of participants by the staff was also mentioned as a contributing factor to program retention*.* Among the most important barriers to participation was the fear of losing custody of or visitation rights to children, male partner jealousy and violence, and experience of discomfort in the NEP waiting area. Suggestions for improvements included additional SRHR services, a “women-only” NEP, as well as improved physical access to NEPs.

### Facilitators for participating in the NEP

This study shows that easy access to other healthcare services within the same site is crucial and a major facilitator for WWID to participate in the NEP. In particular, SRHR services such as contraceptives, cervical cancer screening, STI-testing, abortion counselling and general counselling were highlighted as important services. This finding corroborates previous studies that emphasize the importance of access to a wide range of additional health services for WWID within HR-programs to increase the level of participation [18, 23]. For example, that easy access to sterile injecting equipment and healthcare services increased harm reduction strategies among PWID was concluded in a meta-synthesis in 2014 [28].

A Canadian study similarly reports that needle syringe programs act as a gateway to other services and constitute a preferred site to receive primary health care among PWID [29]. Another study demonstrates how needle syringe programs can act as ‘trusted sites’ and further improve uptake of other health-related services among WWIDs [28]. The findings of this study further support these claims. Consequently, this study make a strong case for recommending add-on services to NEP, including health and welfare services. Therefore, to offer WWID specific services, particularly SRHR services, should be further implemented and evaluated as a part of harm reduction facilities such as NEP.

Supporting previous research, the results of this study also demonstrate how the NEP staff’s caring approach significantly contributes to continued participation [30]. Previous experiences of stigma from being a person who use drugs were described as partially overcome by how the staff at the NEP treated the participants, i.e. with encouragement and care. Trust in the staff and the staff’s ability to create a safe environment has previously been shown to be an important contribution to maintaining participation in NEP [19]. Positively expressed attitudes among staff towards PWID has previously shown to significantly contribute to successful programs as well as maintained participation [29–31]. For instance, a study evaluating Canada’s first women-only low threshold drug consumption site, suggests in accordance with this study’s results, that the staff’s caring approach is important for participation [30].

### Barriers to participating in the NEP

The perceived risk of being reported to social services constitutes a large barrier for participation among WWID who are mothers. Another recent study, which interviewed WWID at a drug consumption site found that their friends and aquinted with children never visited. The WWID interviewed concluded that they had never seen the women they know with custody of children coming to the drug consumption site, in fear of repercussion and child apprehension [30]. Moreover, this study shows that few of the positive aspects typically associated with NEP were seen to compensate for this barrier.

Several studies from the Canadian setting also report that involuntarily child removal due to child protection services´ actions induces increased mental health disorders including post-traumatic stress disorder and poorer health among WWID [32, 33]. In addition, increased alcohol and drug consumption to numb the pain of separation, a higher vulnerability to housing instability and initiation to sex work has been reported [32]. The literature stresses the importance of family support programs for WWID, to support those who has maintained custody as well as WWID who has lost custody of their children [32]. However, the balance between WWID children’s welfare and rights, parental rights and HR is a challenging subject for an ongoing discussion.

Suspected low levels of knowledge among the participants of this study regarding the legal procedures related to involuntary child removal due to child protection may contribute to increase the concern. This issue can be addressed by the Stockholm NEP by working more actively to fill the knowledge gap and prevent misinformation. Moreover, further studies on WWID with children and the different ethical and legal aspects are needed in order to reach these women.

Participants who were subjected to their male partners’ jealousy and violence raised this as an additional barrier to NEP participation. In this study, male partner jealousy was expressed as an issue particularly in the context of a perceived risk for the woman meeting other men in the waiting area. Moreover, participants reported that they did not feel safe in the waiting area due to the behaviour of other male participants such as harassment (including sexual harassment) and the risk of meeting perpetrators of physical and psychological abuse. This is supported by previous research, which has suggested that WWID may not use NEP due to fear of violence [34]. In Canada, women using the women-only facility described a significantly different experience compared to using the mix-gendered facility, in regards to feeling safer and being able to escape violence at the women-only facility [30].

Several studies have investigated and concluded that gendered-based violence decrease the access for WWID to HR-services [30, 35, 36]. Normalizing violence as a part of every-day life for WWID is not uncommon, one study describe how WWID are injecting alone to a greater extent, thereby minimizing the risk of gendered violence [36]. The need for gendered-attentive HR-interventions is evident and further strengthened by our findings [36]. There is a need to further investigate how gender-based interventions are best implemented to reduce violence and increase WWIDs´ access to HR-services, and what services that could be implemented as part of NEP. A possible intervention to overcome this barrier could be the introduction of a women-only NEP in Stockholm, or offering exclusive opening hours for women, as suggested also by the study participants and previous studies [21, 22, 30].

Being identified as a person who use drugs was raised as an additional barrier to coming to the NEP. Participants expressed that visiting the NEP could initially reinforce the stigma and feeling of shame related to being a person who use drugs [24, 37]. However, after joining the program, the feeling of stigma reportedly decreased due to the trust participants felt in the staff, as previously reported from NEP services in Sydney, Australia [19]. Fear of being registered in the medical chart and by social services was also evident in the analysis. Similar data have been reported from other settings, and anonymous participation in NEP is therefore recommended as an international standard to facilitate NEP access and uptake [31]. Our results suggest that this recommendation is also applicable in a Swedish setting.

Fear of a positive test result due to mandatory testing for HIV and hepatitis was also raised by participants as a barrier for NEP participation. However, participants expressed that this barrier was overcome when staff informed them of that treatment and support was readily available in case of a positive test result. HIV and hepatitis testing and treatment is one of the main assignments of NEP programs globally in order to reduce the burden of these diseases [15]. Therefore, increasing the access of information to potential participants who are not yet in the program, especially WWID, regarding for example available treatment options at the NEP may be a way to overcome this barrier. Information may be organized via OST clinics or other healthcare units.

In this study, practical barriers that were identified concerned both opening hours and geographical distance to the NEP, which are consistent with previous studies’ findings [28]. To increase accessibility, mobile units have been implemented successfully in several settings and proven valuable to increase access to HR-services and reduce the spread of HCV and HIV [38, 39]. While smaller mobile units may increase uptake of sterile injection equipment, add-on health interventions for women such as SRHR services might be logistically difficult to implement at such units. In addition, WHO stated that the most successful NEPs are those with a multitude of services and different methods to access PWID, for example mobile units [31]. Already in 2007, WHO recommended that additional health services should be implemented at existing NEP sites [31].

Consistent with the results in this study, increased accessibility would likely increase WWID participation. Furthermore, to facilitate further uptake amongst WWID, extended opening hours, including evenings and weekends, would be recommended based on the results in this study. Additional NEP sites and mobile units would also likely facilitate uptake.

### Suggestions for improvements of the NEP

Improvements of the NEP included suggestions regarding NEP only for women as well as opening hours solely for women. Targeted NEP services for women in other settings have been shown to facilitate uptake among WWID [40, 41]. Further, a study focusing on WWID in the UK and Australia reported that women-only NEP resulted in higher uptake among WWID, while at the same time implied a potentially negative effect of adding to the perceived stigma among WWID [42]. However, another recent study showed that WWID felt that the staff-approach and the women-only environment lessened the perceived stigma [30].

Another study which investigated if WWID preferred women-only services in the context of substance use disorder treatment facilities, concluded that not all women requested women-only services beforehand, but those who did reported positive to the experience [42]. A recent review on the effectiveness of drug treatment programs for women only found that this approach increased retention in treatment [22]. Whether this is translatable to NEP services remains to be investigated. Based on the data gathered during this research together with previous studies, one suggestion would be to provide women-only opening hours in existing NEP as a first step.

Targeting women’s specific needs regarding healthcare services may further improve NEP uptake. Participants particularly requested SRHR services and education. Previously suggested by other studies, this study thus concludes that these interventions should be implemented and evaluated within NEP programs [21, 22, 28].

### Limitations

This study has some limitations. First, only WWID who were already participating in the NEP were recruited to the partake in the study. As a result, the study does not capture the experiences regarding barriers to participation among non-participants. Furthermore, the group of WWID who agreed to participate may be a biased sample, representing those who have had a positive experience of the NEP. Whilst acknowledging these two limitations, it is important to highlight that all participants shared sensitive information about their lives indicating that trust was established.

## Conclusion

The present study provides a significant contribution to previous knowledge regarding WWIDs’ own perspective and needs in relation to NEP participation. More specifically, the results contribute to knowledge regarding factors serving as barriers and facilitators for participating in NEP as expressed by WWID themselves in a region with a history of low coverage of HR interventions. Offering a wider range of services, particularly SRHR services, and increasing access through a “women-only” alternative at existing sites, may serve as sufficient compensation for the identified barriers. These interventions would, according to this study, contribute to the work of scaling up NEP, and to promote and enhance WWID access to healthcare and harm reduction services. Future studies should include WWID not yet using the NEP, to more thoroughly investigate barriers and facilitators for participation.

## Data Availability

The interview transcripts generated during the interviews in this study are not publicly available in order to preserve the confidentiality of the participants.

## Abbreviations

PWID: People who inject drugs
WWID: Women who inject drugs
HR: Harm reduction
NEP: Needle exchange program
SRHR: Sexual and reproductive health services
IDI: In-depth interview
STI: Sexually transmitted infections
HIV: Human immunodeficiency virus
HCV: Hepatitis C
HBV: Hepatitis B
OST: Opiod substitution treatment
HAV: Hepatitis A
